# A Case of Breast Papillary Carcinoma in an Elderly Black Male Patient With Pathology, Ultrasound, and Mammogram Imaging Findings

**DOI:** 10.7759/cureus.66684

**Published:** 2024-08-12

**Authors:** Thomas Kent, Danielle R Petty, Mariam Hanna

**Affiliations:** 1 Department of Radiology, University of Florida, Gainesville, USA; 2 Department of Pathology, Immunology, and Laboratory Medicine, University of Florida College of Medicine, Gainesville, USA; 3 Department of Radiology, University of Florida College of Medicine, Gainesville, USA

**Keywords:** ultrasound-guided core biopsy, bilateral gynecomastia, male breast imaging, male breast carcinoma, breast papillary lesions

## Abstract

The patient was an 84-year-old man who presented with a palpable, left breast mass. Following ultrasound, mammography, and ultrasound-guided core needle biopsy, the lesion was diagnosed as papillary carcinoma. Findings included a complex, cystic mass on ultrasound; a well-circumscribed, high-density lesion on mammogram; and a lack of highlighting of myoepithelial cells within fibrovascular cores on immunostaining. With this case report, we aim to add to the literature an additional example of breast papillary carcinoma in a male patient and its corresponding imaging and pathologic findings.

## Introduction

The incidence of male breast cancer (MBC) is reported to be less than 1% of all breast cancers [1]. In 2023, there are expected to be 2,800 incident breast cancers in men compared to 297,790 in women [2], with lifetime risk estimated to be 1:1,000 for men compared to 1:8 for women [3]. Risk factors for MBC include increasing age; black race; a family history of breast cancer; positive mutations for *BRCA2*, *BRCA1*, *CHEK2*, and *PALB2*; radiation exposure; increased serum estradiol; Klinefelter’s syndrome; gynecomastia; liver disease; obesity; and testicular abnormalities. Overall, 99% of MBC cases are estrogen receptor (ER)-positive compared to 83% of female breast cancer cases. In MBC, the most common histologic type is invasive ductal carcinoma (>80%), followed by ductal carcinoma in situ (DCIS) (10%), and the much rarer subtypes of papillary (2-3%), mucinous (1-2%), and lobular (1-2%), which contrasts with female breast cancer, where lobular and DCIS comprise 12% and 25% of cases, respectively [3]. Papillary carcinoma can be further classified as encapsulated, solid, invasive, intracystic, and papillary DCIS [4,5].

A few case reports have documented imaging findings of papillary carcinoma in male patients, with or without gynecomastia, and of various types [1,6-9]. We report the case of an elderly male patient with bilateral gynecomastia and a unilateral, left palpable breast mass, characterized by ultrasound and mammogram and diagnosed as a low-grade papillary carcinoma.

## Case presentation

An 84-year-old Black male patient with a medical history significant for benign prostatic hyperplasia, left bundle branch block (LBBB), and obesity (body mass index = 33.64 kg/m^2^) presented with breast pain and a palpable left breast mass extending to the retro-areolar region. The mass was discovered by the patient’s son a few weeks before the initial visit at an outpatient primary care provider, who referred him to outside mammography. The patient denied any other palpable masses, nipple discharge, skin changes, or axillary adenopathy and reported no significant history of breast disease or past breast biopsy. Family history was positive for a sister and brother with cancer of unknown type and sisters who died of lung cancer.

**Figure 1:**
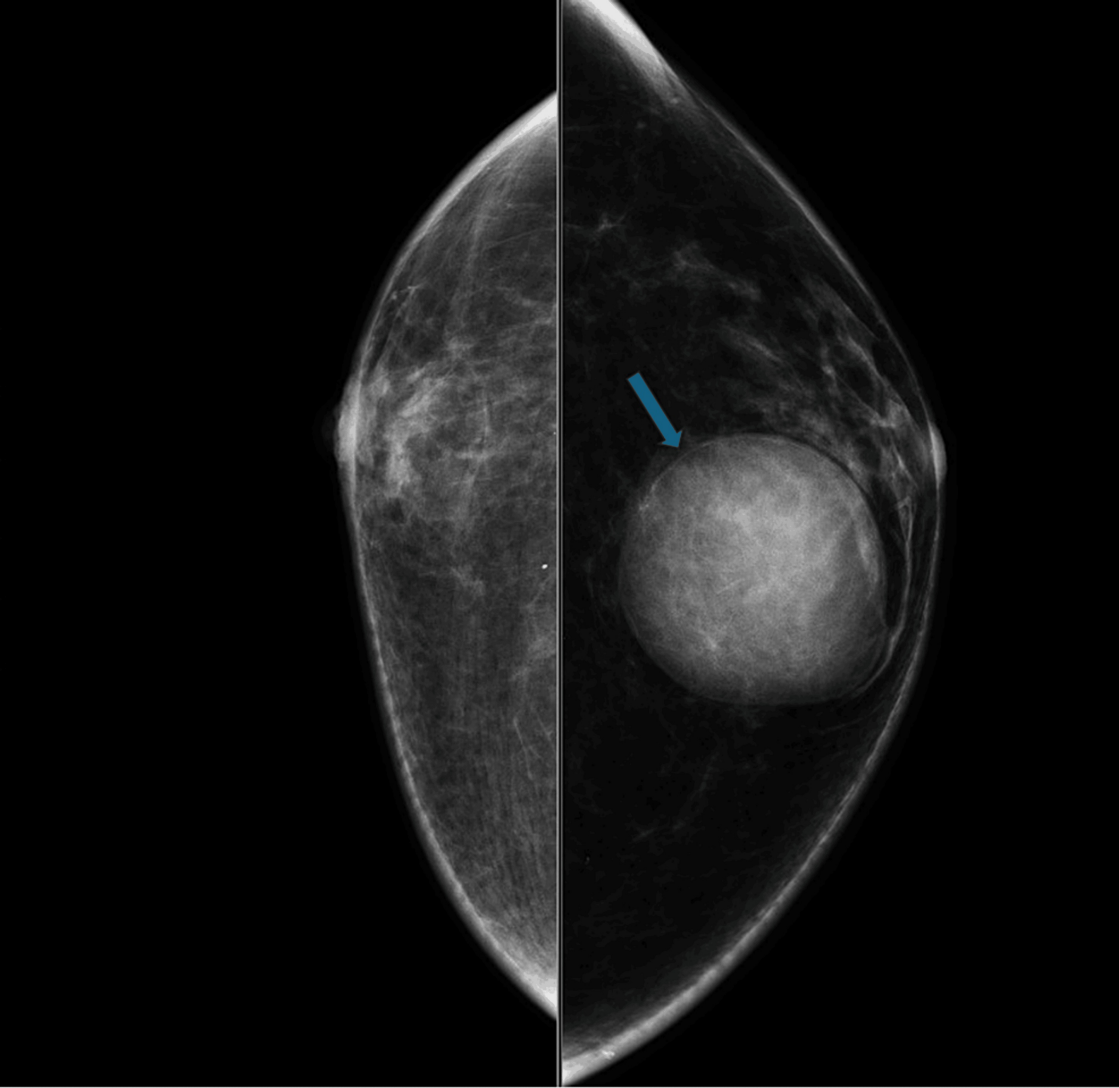
Mammogram. Left breast craniocaudal mammographic projection demonstrating a high-density mass with circumscribed margins in the left subareolar region (blue arrow).

**Figure 2:**
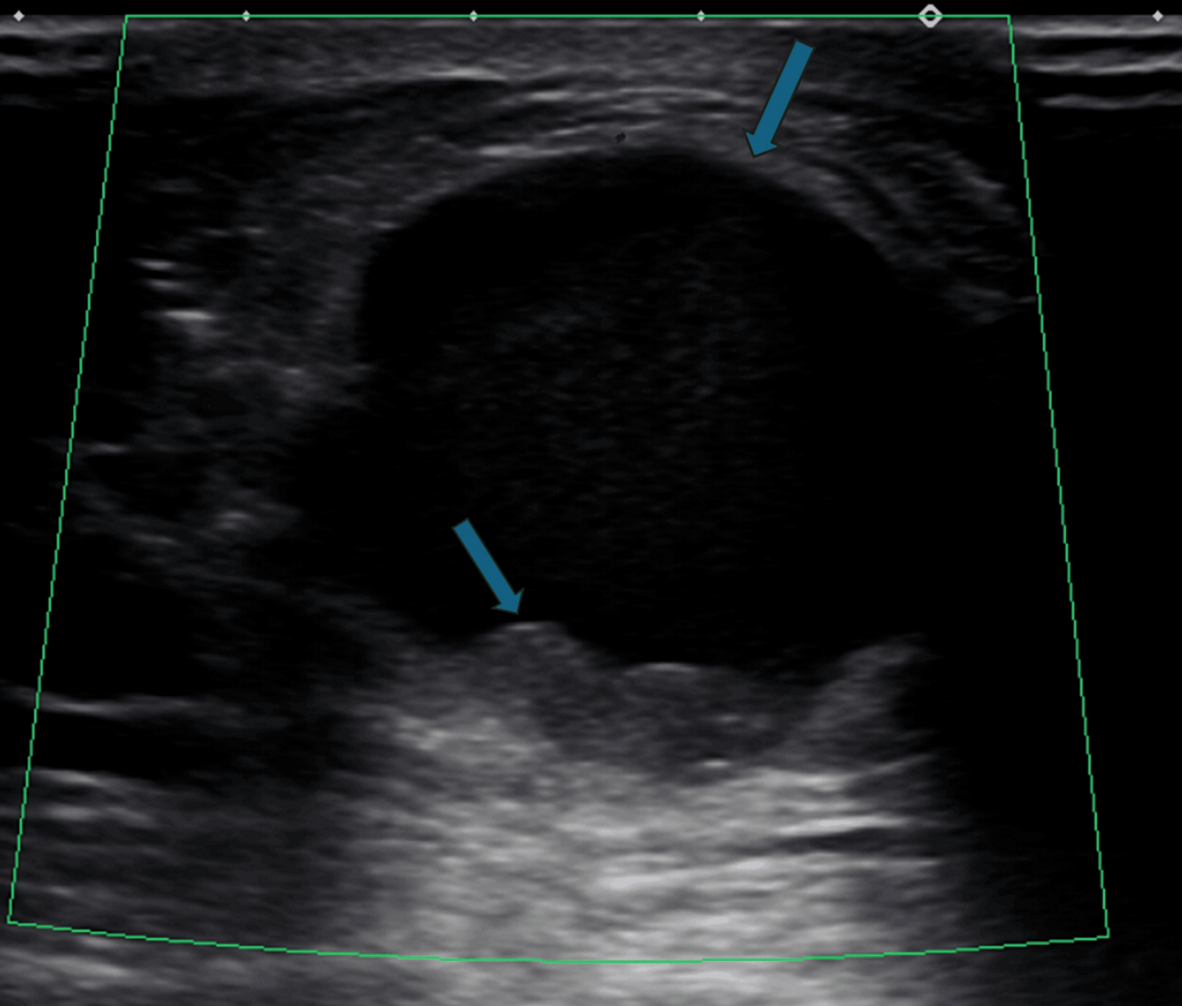
Ultrasound. Grayscale image demonstrating a complex solid and cystic mass in the left subareolar region (blue arrows).

**Figure 3:**
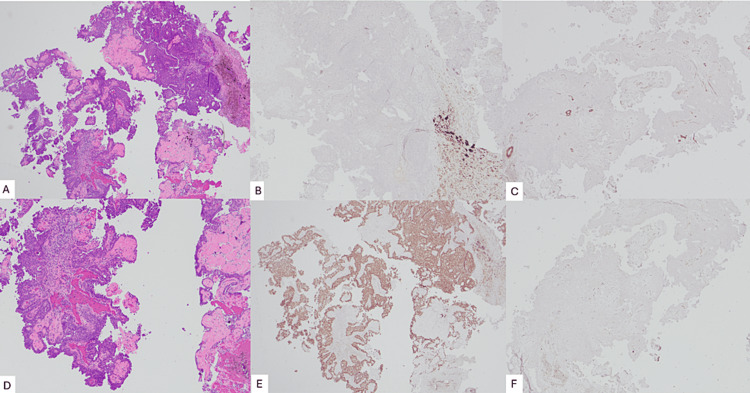
H&E shows a neoplasm with a monomorphic cell population with papillary architecture containing fibrovascular cores. Cytokeratin 5/6 was lost within this cell population, and ER was strongly and diffusely positive, consistent with a clonal cell population. SMMHC and p63 were used to discern the pattern of myoepithelial staining, which can help determine the type of papillary lesion. In a benign papilloma, for instance, myoepithelial cells should stain both at the periphery of the lesion and along the lining of the fibrovascular cores. In this case, however, both stains were predominantly negative along the periphery and the fibrovascular cores, most in keeping with an encapsulated papillary carcinoma or solid papillary carcinoma. A. Hematoxylin and eosin (H&E); 20×. B. p63 immunohistochemistry (IHC); 20×. C. Smooth muscle myosin heavy chain (SMMHC) IHC; 20×. D. H&E; 40×. E. Estrogen receptor (ER) IHC; 20×. F. Cytokeratin 5/6 IHC; 20×.

The outside mammogram revealed mild bilateral gynecomastia and a large, circumscribed mass in the 12:00 area of the left breast, corresponding with the palpable mass (Figure 1). Subsequent whole breast demonstrated the mass as complex, with a size of 3.6 × 3.1 × 3.5 cm, and structure primarily cystic, but with a solid component along the periphery (Figure 2). No axillary lymphadenopathy was noted. The mass was categorized as BI-RADS 4 with excision recommended. The patient then presented to our institution, where a repeat mammogram and ultrasound confirmed these findings. An ultrasound-guided core needle biopsy was performed via an ATEC vacuum-assisted 12-gauge biopsy device with multiple biopsy samples sent to pathology for analysis. Following the biopsy, there was a resolution of the mass’s cystic component on ultrasound. Grossly, the tissue was glistening and tan-yellow. Microscopy demonstrated a fragmented neoplasm with a papillary growth pattern and low-grade, monomorphic cytology, with no overtly invasive component identified. Immunohistochemistry was diffusely and strongly positive for ER with CK5/6 lost. Smooth muscle myosin heavy chain (SMMHC) and p63 were patchy but did not highlight myoepithelial cells within the fibrovascular cores of the lesion (Figure 3). Further characterization of the mass was deemed possible following total resection, with left mastectomy being the preferred treatment. Subsequent genetic testing of 31 genes, including *BRCA1*, *BRCA2*, *CHEK2*, and *PALB2*, were negative except for a mutation in the *ATM* gene of unknown significance. Left mastectomy was postponed following abnormal EKG results consistent with LBBB.

## Discussion

The differential diagnosis for a palpable breast lesion in a male includes gynecomastia, lipoma, intraductal papilloma, sebaceous cyst, and MBC, among others. MBC is a rare entity, and papillary carcinoma of the male breast is rarer still. Papillary carcinoma, a neoplastic proliferation of cells with fibrovascular stalks without an intact myoepithelial layer, has a higher incidence in men than women [6].

Preventative mammography screening for MBC is not recommended, and, as a result, DCIS, which is not typically palpable, is not commonly discovered. With a later diagnosis, most MBCs are invasive carcinomas, with a palpable mass being the presenting complaint, as in this case [3]. Of the risk factors for MBC, our patient was positive for Black race, elderly age, obesity, and gynecomastia. His family history was positive for lung cancer and other unknown cancers, which cannot be commented upon.

In MBC, the incidence rate is 52% higher in Black men than in white men, particularly when ER is positive, but the reasons are largely unknown [10]. The connection between gynecomastia and MBC is also largely undetermined, with some studies showing the incidence of gynecomastia in MBC patients to be at or lower than the overall baseline population level, suggesting a tenuous link [7]. The small number of MBC cases prevents conclusions one way or the other. Gynecomastia itself is an indication of a high estrogen-to-androgen ratio, like the implication of several other MBC risk factors, such as liver disease, obesity, and testicular abnormalities.

Ultrasound imaging and mammography are important tools to distinguish between gynecomastia and MBC, which can sometimes be subtle. On ultrasound, bilateral, disk-shaped, hypoechoic retro-areolar tissue is consistent with gynecomastia while discrete masses, cystic components, or complex appearance are not and are suspicious for malignancy. On a mammogram, a fan-shaped central subareolar opacity is consistent with gynecomastia, while an irregular, spiculated lesion, unilateral involvement, eccentric location outside the nipple-areolar complex, and higher density than background is suspicious for malignancy [3,11].

In papillary carcinoma, the typical ultrasound findings include a complex heterogenous mass formed by solid and cystic components. On mammography, papillary carcinoma appears as a subareolar mass that is variable in shape, including circumscribed, oval, lobulated, or irregular, with irregularity suggestive of invasion [11].

In this case, imaging was consistent with a papillary carcinoma, given the findings of a unilateral, complex, cystic structure with a solid component on ultrasound (Figure 2), and a circumscribed, sub-areolar mass on mammogram (Figure 1). The background of bilateral gynecomastia did not confound the diagnosis in this case, as the mass was apparent with distinct borders and an eccentric location. While pathologic analysis did not find an overt invasive component, the lack of highlighting of myoepithelial cells within fibrovascular cores, by immunostaining for p63, SMMHC, and CK5/6, was not re-assuring (Figure 3), which is the main objective criterion to distinguish in situ from invasive carcinoma [7].

Regarding prognosis, men with breast cancer have similar survival rates as women after adjusting for age at diagnosis and shorter male life expectancy. Black men with breast cancer have similar rates of survival as white men after adjusting for income and insurance coverage. In terms of treatment, most men undergo mastectomy, although endocrine, radiation, and breast-conserving therapies can also be used [3]. Importantly, MBC survivors have a higher risk of second primary cancers [6].

## Conclusions

In this case report of MBC, ultrasound revealed a complex, cystic mass; mammography demonstrated a high density, circumscribed mass; and ultrasound-guided core needle biopsy with subsequent staining was consistent with an encapsulated papillary carcinoma or solid papillary carcinoma. MBC is a rare entity, with relatively few documented cases, and, unfortunately, often presents at a later stage due to a lack of general preventative screening. In this case, the patient’s son initially identified the lesion. Gynecomastia is not necessarily a causative factor but may indicate the presence of other risk factors associated with higher male estrogen levels. In the future, perhaps new genetic or other markers will be identified which will enable screening for MBC in high-risk male patients.
